# Medical handovers in the ICU: a snapshot of practice in the South West of France

**DOI:** 10.1186/cc9911

**Published:** 2011-03-11

**Authors:** G Brenier, T Geeraerts, O Fourcade

**Affiliations:** 1 CHU Toulouse Prupan, Toulouse, France

## Introduction

Medical handover is critical for quality of care in the ICU. Time assigned to medical handovers can vary across different units, with significant impact on the organization of medical work. We aimed to study the time spend for medical handover in ICU and its variation across academic, general and private hospitals in the area of the South West of France, the Midi-Pyrénées region.

## Methods

Between August and October 2010, we questioned by telephone 86 physicians issued from 19 different ICUs. This prospective observational study mainly focused on four items: unit characteristics, health diary organization, medical handover procedures, and self-assessment of satisfaction for medical handover (numeric scale from 0 to 10).

## Results

**Figure 1:**
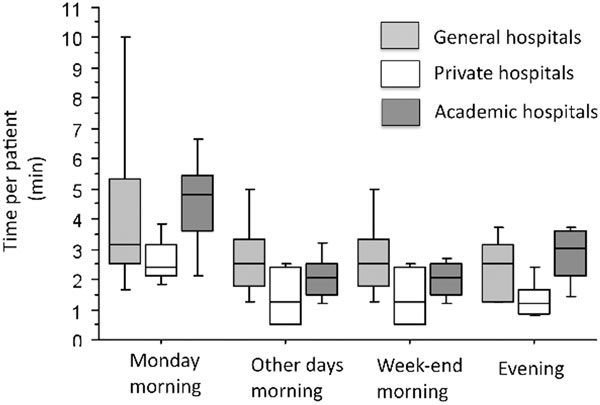


Eleven general hospital centers, three private hospitals, and five university hospitals were concerned by the survey. The mean time spent for medical handover was 59 ± 35 minutes on Monday morning, significantly longer than other days, evening, and weekend handovers (*P *< 0.001 for all comparisons). When reporting it with the number of ICU beds, the time spent for handover per patient was significantly shorter in private hospitals compared with general and academic hospitals (*P *< 0.05 for all comparisons). This was true for every day. The median satisfaction for quality and duration were both 8, with a significantly higher satisfaction in general hospital (*P *= 0.001 for comparison vs. other hospital for both). See Figure 1.

## Conclusions

Time spent for the medical ICU is important, with an approximate total time of 1 hour 30 minutes on Monday, and 1 hour the other days. Physicians in private hospitals spend less time for medical handovers. This fact should be considered for medical timework organization, especially in academic hospitals and in hospitals with large ICUs.

